# Internetberatung und Psychotherapie: reflektierte Erfahrungen von Psychotherapeut_innen und Klient_innen

**DOI:** 10.1007/s00729-021-00186-1

**Published:** 2021-12-07

**Authors:** Margarete Finger-Ossinger

**Affiliations:** 1 Personenzentriertes Lernen, psychosoziale Bildung und Weiterbildung,, Schützengasse 25/1und 5, 1030 Wien, Österreich; 2 Forensische Ambulanz, Weingartshofstraße 37, 4020 Linz, Österreich

**Keywords:** Tele-Psychotherapie, Covid19-Krise, Optimale Behandlung, Individualität, Tele-psychotherapy, Covid19 crisis, Optimal treatment, Individuality

## Abstract

Internetberatung ist in Österreich durch die Internetrichtlinie für Psychotherapeut_innen festgelegt, jedoch erlaubt diese nicht die psychotherapeutische Behandlung mittels Medien. Es wird darauf hingewiesen, dass derzeit keine Psychotherapie via Internet eines wissenschaftlich begründeten und evaluierten Vorgehens existiert. Interessen dies zu ändern, gibt es schon seit Jahren; die Herausforderung des Lockdown in der Covid19-Krise haben es – zumindest zeitbeschränkt – möglich gemacht, psychotherapeutische Behandlung mittels Medien durchzuführen.

Die reflektierten Erfahrungen, die Psychotherapeut_innen und Klient_innen mit dieser ausnahmsweise gewährten Form von Psychotherapie gemacht haben, können ein kleiner Beitrag dazu sein, herauszufinden, ob Psychotherapie – weitere Forschung vorausgesetzt – auch mittels Internet oder Telefon stattfinden sollte, welche Faktoren zu beachten sind oder ob optimale Krankenbehandlung immer unmittelbar, im engeren Verständnis dieses Wortes, fachlich vertretbar ist.

## Abstract (en)

Internet counseling is defined in Austria by the Internet guideline for psychotherapists, but this does not allow psychotherapeutic treatment via media. It is pointed out that currently there is no psychotherapy via internet of a scientifically founded and evaluated procedure. Interests to change this have existed for years; the challenge of the lockdown in the Covid19 crisis have made it possible—at least time limited—to conduct psychotherapeutic treatment via media.

The reflective experiences that psychotherapists and clients have had with this exceptionally granted form of psychotherapy can be a small contribution to finding out whether psychotherapy—assuming further research—should also take place by means of the Internet or telephone, which factors are to be taken into account or whether optimal treatment of the sick is always directly professionally justifiable.

## Einleitung

Wie das Datum des Psychotherapie Forums, vol. 13, Suppl. 2, Nr. 2005, S. 43ff zeigt, in dem die Richtlinie des Bundesministeriums für Gesundheit (BMG 2005) veröffentlicht wurde, beschäftigte das Thema Beratung oder Psychotherapie per Internet zu ermöglichen die Psychotherapieszene schon vor Covid19. Die derzeit gültige Internetrichtlinie ist das Ergebnis eines mehrjährigen Diskussionsprozesses, insbesondere im Rahmen des Psychotherapiebeirates. Eine grundlegende Überarbeitung ist aufgrund der zunehmenden Digitalisierung sicher dringend notwendig geworden.

Die Internetrichtlinie schließt derzeit dezidiert die Behandlung via Internet aus und verwehrt sich auch gegen die Anwendung missverständlicher Begriffe, wie „Online-Therapie“ oder „virtuelle Couch“. Sie äußert sich zur psychotherapeutischen Beratung wie folgt:

*1.1 Ausschluss der psychotherapeutischen Behandlung*

*Bei der Beschreibung des Phänomens „Psychotherapie und Internet“ ist zu berücksichtigen, dass derzeit keine Psychotherapie via Internet im Sinne eines wissenschaftlich begründeten und evaluierten Vorgehens existiert und in der Folge die Verwendung von Begriffen, wie etwa „Cyber-Therapie“, „Online-Therapie“ oder „virtuelle Couch“ durch Psychotherapeutinnen und Psychotherapeuten zum Zwecke der Beschreibung von Leistungsangeboten im Internet sowohl aus fachlicher, berufsethischer und berufsrechtlicher Sicht abzulehnen sind*. (…)

*2.1 Verpflichtung zum Erwerb spezifischer Kenntnisse und Erfahrungen in der psychotherapeutischen Beratung*

*Zu beachten ist, dass sich der Psychotherapeut gemäß § 14 Abs. 5 PthG bei der Ausübung seines Berufes auf jene psychotherapeutischen Arbeitsgebiete und Behandlungsmethoden zu beschränken hat, auf denen er nachweislich ausreichende Kenntnisse und Erfahrungen erworben hat. Psychotherapeutische Beratung via Internet ist jedenfalls als eigenes psychotherapeutisches Arbeitsgebiet zu qualifizieren, weil beträchtliche Unterschiede zur herkömmlichen psychotherapeutischen Face-to-Face-Beratung und -Behandlung bestehen*. (…)

*Die Schwelle einer psychotherapeutischen Beratung zu einer psychotherapeutischen Behandlung via Internet wird jedenfalls dann überschritten werden, wenn in einer Summenbetrachtung die Indikation für eine psychotherapeutische Behandlung gegeben ist. Dies wird insbesondere dann der Fall sein, wenn der Verdacht einer krankheitswertigen Störung auftritt und die psychotherapeutische Beratung im Hinblick auf Setting, Gegenstand und Ziel der Beratung und die angewandten methodischen Instrumente die Rahmenbedingungen einer psychotherapeutischen Behandlung annimmt* (BMSGPK 2020).

Trotzdem die Internetrichtlinie versucht zwischen Beratung und Psychotherapie zu unterscheiden, sich im Untertitel auch nur auf psychotherapeutische Beratung bezieht und dies vor allem auch an der „Krankheitswertigkeit“ festmacht, ist bei den Angeboten im Internet für Konsument_innen schwer unterscheidbar, ob es sich bei den Angeboten um Beratung oder Psychotherapie handelt. Der Begriff *„psychotherapeutische Beratung“* kann zur Verwirrung führen. Werden die Schlagwörter „*Online-therapie*“ und „*Österreich*“ in eine Suchmaschine eingegeben, so findet sich zum Beispiel zuerst eine deutsche Seite mit der Headline „*Online Psychotherapie Österreich – Rund um die Uhr verfügbar*“. Hier wird „Therapie vor Ort“ und Beratung per Internet verglichen und letzteres nicht nur in der Beschreibung („*sofort verfügbar“, „anonym“, „billiger“ und „ohne Anfahrtswege verfügbar*“), sondern auch farblich so beworben, dass „Therapie vor Ort“ als wenig attraktiv erscheint. (Insta Communications GmbH 2015/2021). Da der Unterschied von Psychotherapie, Therapie und psychologischer Beratung per Internet für Konsument_innen schwer zu erkennen ist, und auch aufgrund der Headline eine österreichische Seite erwartet wird, stellt sich die Frage, inwieweit der Konsumen_innenschutz gewährleistet ist.

Bereits vor dem ersten Lockdown in der Covid19-Krise im März 2020 wurden von Psychotherapeut_innen in Österreich in Informationsveranstaltungen die Vorteile von „*psychotherapeutischer Beratung via Internet“* propagiert, ohne klarem Hinweis auf die Abgrenzung zu „*Psychotherapie“* und Verweis auf die Internetrichtlinie, die sich nur auf „*psychotherapeutische Beratung“* bezieht. Werbewirksam und engagiert wurden und werden virtuelle Praxisräume angeboten und die Vorteile „unterschiedlicher Formen der Online-Beratung“ (Legerer-Bratengeyer et al. 2021) aufgezeigt. Online-Beratung und Psychotherapie werden als quasi gleichwertig oder sich ergänzend dargestellt. Die aktuelle Beschreibung einer Weiterbildung, die auch schon in etwas anderen Worten vor der Covid19-Krise beworben wurde, lässt das übliche Setting fast alt aussehen. So heißt es etwa „Digitalisierung findet auch in der Psychotherapie statt“, „ganzheitliche und moderne Strategien“, „neue Form der Begegnung“, „synchrones Setting in der Videosprechstunde“, Ziel sei es die unterschiedlichen Formen der Online-Beratung ergänzend zum *„f2f Setting“* in den Praxisalltag aufzunehmen (ebd.).

## Dann kam Covid-19

Angestoßen durch die Ausgangsbeschränkungen im ersten Lockdown der Covid-19 Krise bemühte sich der Berufsverband für Psychotherapie auf Bundes-, und Landesebene bei der Österreichischen Gesundheitskasse um eine Ausnahmeregelung für die Fortführung von Psychotherapien. Da soziale Kontakte wegen des unbekannten Virus minimiert werden sollten, gestatteten die Krankenkassen in dieser Ausnahmesituation bei Bedarf die Überbrückung der Psychotherapien durch Sitzungen per Telefon oder Internet. Diese Möglichkeit widerspricht zwar der Internetrichtlinie des BMSGPK, jedoch weist der ÖBVP darauf hin, dass „Sollte ein/e PatientIn jedoch dringend *psychotherapeutische Unterstützung* (Hervorhebung die Autorin) benötigen, stellt der Einsatz elektronischer Medien zur Überbrückung einer Notlage eine Möglichkeit dar, sofern der/die PatientIn andernfalls einen Schaden erleiden könnte“. (Wiener Landesverband für Psychotherapie 2021).

Aufgrund der nun bereits mehr als einjährigen Erfahrung dieses Angebotes und einer Umfrage der Donau-Universität Krems und des ÖBVP unter 1800 Psychotherapeut_innen, die eine „deutlich positivere Erfahrung als erwartet“ mit der Therapie per Telefon oder Videochat aufzeigt, wird die Forderung erhoben, die „Erfolgsgeschichte der Tele-Psychotherapie“ weiterzuschreiben und die Angebote auch nach dem Ausnahmezustand der Pandemie weiterzuführen. Das Behandlungsverbot via Telefon und Internet sei aufzuheben um auch eine „breitflächige Versorgung“ der Patient_innen „zu jederzeit und unter allen Umständen“ (NOE ORF 2021) zu gewährleisten.

Rasch auf neue Herausforderungen zu reagieren, wird in der Regel mit Aktivität, Jugendlichkeit, Anpassungsfähigkeit und Flexibilität assoziiert, während Langsamkeit mit Alt sein, Starrheit und Inaktivität gleichgesetzt wird. Psychotherapie ist ein junger Berufszweig und herausgefordert, sich im Gesundheitswesen zu profilieren. Die meisten Psychotherapeut_innen möchten nicht „alt aussehen“, obwohl sie im Gegensatz zu Patienten_innen der Einbeziehung von Medien anfangs eher skeptisch gegenüberstanden. „Umso erfreulicher ist es, dass die anfängliche Skepsis der Therapeuten schnell überwunden war“, so Barbara Haid, Präsidiumsmitglied des ÖBVP (ebd.).

Am 17.11.2020 wurde von den Abgeordneten Gerald Loacker und Douglas Hoyos-Trautmannsdorff, sowie nicht genannten Kolleginnen und Kollegen ein Entschließungsantrag eingebracht: Der Nationalrat wolle beschließen:

*Die Bundesregierung, insbesondere der Bundesminister für Soziales, Gesundheit, Pflege und Konsumentenschutz, wird aufgefordert, zu evaluieren, inwiefern bzw. in welchem Umfang die bestehende Rechtslage Psychotherapie mittels Telemedizin (Online-Psychotherapie) erlaubt und diese gegebenenfalls entsprechend zu adaptieren.* (Loacker und Hoyos-Trautmannsdorff 2020).

## Cui bono?

Selbstverantwortliche Psychotherapeut_innen sind jedenfalls gefordert, ethisch begründet, die Entscheidung zu treffen, und sich in (politische) Diskussionen einzubringen, weshalb, inwieweit, in welcher Form, für welche Patient_innen eine Psychotherapie mittels Medien (sollte diese ihrem Wesen nach gleichzuhalten sein) gerechtfertigt sein könnte. Da Psychotherapeut_innen ihren Beruf nach bestem Wissen und Gewissen und unter Beachtung der Erkenntnisse der Wissenschaft ausüben sollen, brauchen Sie auch für die Zukunft eine hinreichende Entscheidungsgrundlage, die möglichst viele Aspekte mit einbezieht.

## Ein Thema – viele Meinungen

Da sich beim Hinhorchen auf verschiedenste Meinungen, Wahrnehmungen und Aussagen der Psychotherapeut_innen und Klient_innen vordergründig ein eher buntes Bild ergab, entstand das Interesse, ob sich gewisse Faktoren finden ließen, die eine weitere Diskussion und Forschung, so wie auch in der Internetrichtlinie gefordert, vorantreiben könnte. Es wird in dieser Studie im Sinne der qualitativen Forschung versucht, offen auf das Thema zuzugehen (Lamnek 2005). Dabei wird auf eine Hypothesenbildung am Beginn verzichtet. Qualitative Forschung versteht sich als Hypothesen generierendes Verfahren. Die Involviertheit des Forschers ist konstitutiver Bestandteil der Forschungsprozesses. Im Rahmen der Podiumsdiskussion der österreichischen Psychotherapieforschungstagung der Gesundheit Österreich GmbH im Oktober 2020 wurde das Anliegen der vorliegenden Arbeit präsentiert und im Tagungsband skizziert.

## Vorüberlegungen

Um möglichst wenig Einfluss auf spontane Einfälle der Interviewpartner_innen zu nehmen, und auch um Beeinflussung durch die Interviewende hintanzuhalten, wurde das episodische Interview gewählt. Diese Interviewform kombiniert Befragung und Erzählung; es handelt sich um eine gesteuerte Narration. Ziel des episodischen Interviews ist es, sowohl semantisches als auch episodisches, also situativ gestütztes Wissen von Interviewpartner_innen zu erfassen. Das episodische Interview lässt sich in verschiedenen Bereichen der Forschung, in der Sozialarbeit und in verwandten Feldern einsetzen, in denen es um Wissen und Erfahrungen in subjektiver Perspektive geht. Subjektives Wissen über einen bestimmten Bereich wird in der qualitativen Forschung in der Regel über Interviews mit mehr oder minder offenen Fragen erhoben (Flick 2006).

Die interviewten Psychotherapeut_innen stammen aus dem beruflichen Umfeld der Behandlerin. Die Auswahl der Klient_innen erfolgte aus dem gesamten aktuellen Sample der Behandlerin. In der qualitativen Forschung sind, anders als in der quantitativen Methodologie, Umfang und Merkmale des Forschungsobjekts unbekannt. Auch die Stichprobengröße ist nicht definiert. Es interessiert weniger, wie ein Problem statistisch verteilt ist, sondern welche Probleme es tatsächlich gibt und wie sie beschaffen sind (Lamnek 2005). Es wird angenommen, dass je strukturierter die Technik der Datensammlung ist, desto unwahrscheinlicher sei das Auffinden neuer Fakten (ebd.).

## Datenerhebung

### Psychotherapeut_innendaten

Es wurden 14 Psychotherapeut_innen bzw. 1 Psychotherapeut_in in Ausbildung unter Supervision interviewt.

Die offene Impulsfrage war in allen Interviews: „Haben Sie/hast du während der Covid19-Krise Erfahrungen mit mediengestützter Psychotherapie gemacht und können Sie/kannst du Unterschiede zum üblichen persönlichen Setting für sich/dich feststellen?“ In Folge wurde der freien Assoziation der Interviewpartner_innen gefolgt.

5 Psychotherapeut_innen boten auch während des Lockdowns ausschließlich Psychotherapie im üblichen Setting mehr oder minder unter den geforderten Sicherheitsvorkehrungen an und wurden deshalb mangels Erfahrung mit Medien nicht in die Studie miteingeschlossen. Von den verbleibenden 10 Psychotherapeut_innen gaben 6 an, Erfahrungen mit Psychotherapie via Zoom gemacht zu haben. 8 Psychotherapeut_innen sammelten Erfahrungen mit Telefonkontakten. Zwei der 10 Psychotherapeut_innen (Tab. 1) bieten bis dato vorrangig Psychotherapie mittels Medien an.

**Table 1:** 

PT/Alter/fmd	Methode	Face to face	Face to face + Medien	Medien
1/55/f	PP	–	x	–
2/64/f	KIP	x	–	–
3/54/f	PP	–	x	–
4/30/f	PP	x	–	–
5/60/m	PP	–	x	–
6/41/f	TA	x	–	–
7/65/f	HYP	–	x	–
8/51/f	HYP	–	x	–
9/47/m	EA	–	x	–
10/33/f	EA	–	x	x
11/45/f	VT	–	x	x
12/56/f	PP	–	x	–
13/61/m	PP	–	x	–
14/43/f	PA	x	–	–
15/67/m	PA	x	–	–

Um Informationen von Spezialist_innen der IT zu erhalten, wurden zusätzlich drei Techniker_innen interviewt. Die offene Impulsfrage lautete: „Psychotherapie kann derzeit auch mediengestützt durchgeführt werden. Was fällt Ihnen dazu ein?“

Die Gespräche wurden aufgenommen und transkribiert.

### Klient_innendaten

Die Auswahl der Klient_innen erfolgte aus dem gesamten aktuellen Sample einer Behandlerin, die Klient_innen aufgrund der Covid19-Krise wahlweise face to face, Telefonkontakt oder Kontakt per Zoom anbietet. Es wurde den Klient_innen freigestellt, welches Setting diese wählen wollten. Sie waren zum Zeitpunkt der Untersuchung zwischen 3 Monaten und 2,5 Jahren in Behandlung.

Von den 34 Klient_innen nahmen bisher während der gesamten Covid19-Krise 8 Klient_innen sowohl face to face als auch Internet in Anspruch, 5 blieben auch bei internetgestützter Psychotherapie, obwohl vor allem im späteren Verlauf der Covid-19 Krise auch die Möglichkeit für face to face bestanden hätte. 21 Klient_innen warteten 2020 den strengen Lockdown ab und nahmen erst dann wieder Psychotherapie in Anspruch, um bei jedem weiteren Lockdown wieder zu pausieren.

Die Klient_innen mit unterschiedlichen Diagnosen wurden aus ethischen Gründen nicht interviewt, sondern Aussagen, die quasi nebenbei zum Thema Covid19-Krise und Setting fielen, von der behandelnden Psychotherapeutin registriert und für die Studie dokumentiert (Tab. 2).

**Table 2:** 

Patient*in f/m/Diagnose/Index	Face to face	Tel./Zoom	Alter
*m/F60/Sch*	x	x	28
*m/F60/S*	x	x	35
*m/F60/St*	x	x	30
*f/F60/N*	x	x	55
*f/F32/S*	x	x	42
*m/F32/W*	x	x	58
*f/F33/E*	x	x	60
*m/F42/M*	x	x	23
**w/F32/J**	–	x	60
**w/F41/K**	–	x	32
**w/F43/K**	–	x	55
**w/F50/Z**	–	x	43
**w/F50/K**	–	x	45
w/F60/G	x	–	34
m/F60/O	x	–	45
w/f32/Z	x	–	25
w/f32/Z	x	–	36
m/F32/K	x	–	37
m/F32/L	x	–	36
w/F32/Sch	x	–	48
m/F32/W	x	–	48
w/F32/Z	x	–	49
w/f32/L	x	–	89
m/F33/H	x	–	52
f/F43/O	x	–	19
w/F43/W	x	–	27
w/F42/H	x	–	29
m/F43/D	x	–	33
m/F43/H	x	–	36
w/F43/*P*	x	–	32
m/F43/L	x	–	45
m/F43/M	x	–	59
w/F50/W	x	–	35
m/F01/*P*	x	–	32

## Auswertung

Die Auswertung der Interviews erfolgte mittels thematischer Analyse nach Braun und Clarke (Braun und Clarke 2006). Die thematische Analyse kann in verschiedene Phasen unterteilt werden, die jedoch flexibel handzuhaben sind. Grundlage waren die transkribierten Interviews und Mitschriften, die in einer ersten Phase mehrmals gelesen werden und anschließend Paraphrasen gebildet werden. Anschließend wird nach Motiven und Kategorien gesucht, die wiederum zu Themen zusammengefasst werden. Dabei ist genau darauf zu achten, dass die enthaltenen Daten nicht zu unterschiedlich sind, sodass sie ein gemeinsames Thema abbilden. Manche Themen müssen nach Durchsicht in separate Themen getrennt werden. Dann folgt ein weiterer Prozess in Bezug auf das gesamte Datenset. In der letzten Phase sind alle Textstellen vollständig kodiert. Der Analyseweg und die dabei auftretenden Schwierigkeiten werden protokolliert (ebd.).

## Aussagen der Klient_innen

### Aussagen zu Bereichen, die Klient_innen angesprochen haben, die auf das „normale“ Setting warten wollten

- **Sicherheit**: „Wenn es mir sehr schlecht geht, bin ich doch allein“. „Ich kann mich nicht wirklich fallen lassen; dann kann ich mich auch nicht einlassen“.
- **Distanz zum Problem**: „Ich geh sonst von zu Hause weg und woanders hin.“ „Da ists vertraut und doch nicht meine Umgebung – das ist mir wichtig“.
- **Datenschutz**: „Telefonieren liegt mir nicht – und das Andere ist mir zu unsicher“.
- **Geschützter Raum: „**Daheim habe ich keine Ruhe; da kann immer jemand reinkommen“.
- **Gesehen werden**: „Ich weiß nicht wirklich, ob da jemand auf mich schaut, ob der Therapeut auch wirklich mit seinen Gedanken bei mir ist“.
- **Ablenkung:** „Privat hab ichs mit Zoom probiert. Ich kann mich nicht auf meine Gefühle konzentrieren. Irgendwie bin ich nicht bei mir selbst.“
- **Atmosphäre**: „Die Stimmung ist anders“. „Ich sehe nicht den gesamten Raum“. „Ich möchte meine Kassenstunden nicht dafür verwenden“.
- **Therapiemethode**: „Bewegung geht nicht online; Körperlichkeit bleibt ausgespart“.

### Aussagen zu Bereichen, die Klient_innen angesprochen haben, die zwischenzeitlich mediengestützt arbeiten wollten

- **Kompromiss**: „Es ist möglich es so zu machen, wenn es nicht anders geht“.
- **Flexibilität**: „Es hat beides Vor-, und Nachteile; jetzt passt es mal auch mit Telefon“. „Ich bin froh, dass meine Stunden so weitergehen können“.

### Aussagen zu Bereichen, die Klient_innen angesprochen haben, die weiterhin mediengestützt arbeiten wollen

- **Bequemlichkeit:** „Ich brauche mich nicht anzuziehen; ich kann gleich so sitzen“. „Ich leg die Füße auf den Tisch und trinke meinen Kaffee“.
- **Angst**: „Ich möchte doch lieber weiterhin warten, bis ich persönlich kommen kann, aber momentan geht das nicht; ich möchte niemanden anstecken und nicht angesteckt werden.“ „Ich habe das Gefühl ich muss ersticken, mit der Maske – und in der Bim muss ich sie tragen.“
- **Körperliche Beeinträchtigung**: „Jetzt nach der Operation ist das schon praktisch. Ich könnte ja gar nicht in die Praxis fahren“.
- **Langer Anfahrtsweg: **„Also das ist praktisch, da erspare ich mir doch eine Stunde Wegzeit“

### IT-Spezialist_innen – zusätzliche Anmerkungen

- **Datenschutz:** „Der Datenschutz ist keinesfalls gegeben“ „Wer hört mit? Es gibt keine 100 %ige Sicherheit; selbst in unserer Branche trifft man sich immer wieder persönlich“.
- **Bequemlichkeit: **„Jetzt in der Krise ist das schon praktisch – sonst könnten wir keine Meetings abhalten“.
- **Das Dazwischen fehlt**: „Die Wahrnehmung ist reduziert; das Unterschwellig Vermittelte fehlt“. „Viele von uns setzen sich dann mit einem Glas Bier von den PC, damit wir etwas Gemeinsames neben dem Thema haben.“
- **Qualität des Bildes**: „Ein Monitor ist immer transportierte Wirklichkeit“. „Das Licht ist anders, die Stimme ist anders“. „Der Hintergrund kann frei gewählt werden – es ist fiktive Wirklichkeit“
- **Sinnesqualität**: „Geruch fehlt“; „2 D ist niemals 3 D“; „die Feinheiten der Körpersprache sind nicht ersichtlich“.
- **Finanzielles**.: „Man erspart sich die Wegkosten – ist praktisch“; „Unsere Büros werden nun aufgelassen – wir arbeiten von zu Hause“. „Warum wollen Psychotherapeuten das? Hat das finanzielle Gründe?“

### Aussagen der 13 Psychotherapeut_innen und 2 Psychotherapeut_innen unter Supervision

- **Eigene Fähigkeiten: **„Ich weiß nicht, ob ich ein klares Setting schaffen kann“; „kenne ich mich mit dem Medium genug aus?“
- **Sicherheit: „**Ich weiß nie, ob nicht vielleicht mit dem Handy aufgenommen wird – das verunsichert mich“. „Letzte Woche habe ich mitbekommen, dass der Partner im Raum anwesend war – die Patientin meinte, er wolle auch mal was mitbekommen.“
- **Methode**: „Was kann meine Methode? Ist sie geeignet?“
- **Beratung-Psychotherapie**: „Ganz klar ist mir der Unterschied nicht; gibt es einen Unterschied?“
- **Patientenbezogen**: „Was könnte das veränderte Setting für meine Klient*innen bedeuten?“ „Kann ich jedem Patienten Psychotherapie per Internet anbieten, auch wenn es erlaubt ist?“.
- **Datenschutz**: „Bin nicht sicher, ob ich mich da genug auskenne“.
- **Unaufmerksamkeit**: „Ich habe oft während der Sitzungen was Anderes gemacht“. „Es war ganz schwierig, sich immer auf die Klienten zu konzentrieren“.
- **Bequemlichkeit**: „Ich hab das Gefühl, je länger ich das auch online mache, desto praktischer find ich das“.
- **Finanzielles**: „Eigentlich bräuchte man dann keinen eigenen Praxisraum; das erspart einiges“.
- **Angst/Angstvermeidung**: „Ich geh jedenfalls so lange nicht in die Praxis, bis ich geimpft bin“. „Ich setze jedenfalls keine Maske auf – in der Praxis jedenfalls ohne!“

## Ergebnisse

Folgende Bereiche wurden insgesamt benannt:

- **Sicherheit**: Sowohl für Psychotherapeut_innen als auch Klient_innen (besonders für jene, die das „normale“ Setting bevorzugten) war Sicherheit Thema. Video, Audio mit möglichen Konsequenzen des Datenschutzes, aber auch der Mangel an emotionaler Sicherheit durch nicht vorhandene Realpräsenz wurden erwähnt.
- **Methode**: Ob die Methode geeignet sein könnte, wurde nur zweimal angesprochen (eine Psychotherapeutin in Ausbildung unter Supervision, ein Klient).
- **Sinne**: Das Thema „Sinneswahrnehmung“ wurde von fast allen Interviewten angesprochen. Die Psychotherapeut_innen merkten an, dass z. B. der Geruch, neben der gesamten Körperwahrnehmung, wie Händedruck, fehle (7 von 10). Die gesamtsinnliche Wahrnehmung wurde besonders von Psychotherapeut_innen ausdrücklich vermisst (9 von 10).
- **Finanzielle Aspekte**: Diese wurden nur zweimal angesprochen, wie z. B. Raum-, oder Wegkostenersparnis, Hoffnung auf eine Zunahme an Klient_innen.
- **Veränderungsprozesse**: Situationswechsel, Umgebungswechsel waren besonders für Klient_innen von Bedeutung, die jedenfalls das „normale“ Setting wahrnehmen wollten. Angesprochen wurde auch ein Perspektivenwechsel (Klient_innen und Therapeut_innen) aufgrund des Ortswechsels.

## Diskussionspunkte zu weiterer Forschungstätigkeit

### (Daten)Sicherheit

Eine wesentliche Frage ist die Datensicherheit. Auch wenn diese nicht immer im Fokus zu stehen scheint, wissen besonders jene, die in der IT tätig sind, dass es eine Letztsicherheit nicht gibt. (Anmerkung: im Rahmen einer Werbeveranstaltung des engagierten Anbieters für eine Plattform zur Online-Therapie: „die letzte Sicherheit gibt’s natürlich nicht“). Hierzu bedarf es genauer Aufklärung für Psychotherapeut_innen und Klient_innen, damit diese aufgrund der Datenlage selbst entscheiden können.

Nicht nur in Bezug auf objektivierbarer Datensicherheit stellt sich die Frage der Anwendung von mediengestützter Psychotherapie, sondern auch hinsichtlich der Diagnosegruppen. So merkte z. B. ein Psychotherapeut an, dass er sich mit „normalen Neurotikern“ gut diese Arbeit per Medien vorstellen könne, jedoch sicher nicht mit Personen, die bereits aufgrund ihrer Lebensgeschichte massive Beziehungsdefizite mitbrächten.

### Fachlich optimal

„Die Beurteilung, ob eine hinreichende Entscheidungsgrundlage für eine psychotherapeutische Beratung (sic!) via Internet vorliegt, ist daran zu messen, ob die durch die Distanz zum Patienten verursachte Einschränkung in der psychotherapeutischen Wahrnehmung noch eine fachgerechte Interaktion mit dem Patienten zulässt.“ (BMSGPK 2020). Es stellt sich die Frage, inwieweit es zulässig ist, und unter welchen Umständen vertretbar, Klient_innen eine optimale Behandlung zukommen zu lassen oder zu verweigern.

### Methoden

Auffallend war, dass nur eine Psychotherapeutin ihre Methode als explizit ungeeignet hielt, um mediengestützte Psychotherapie anzubieten. Dazu bedarf es sicher noch einiger Forschungstätigkeit, inwieweit welche Methode, für welche Patient_innen, unter welchen Bedingungen für dieses Setting geeignet ist.

### Klient_innendaten

Das geringe Sample der Untersuchung lässt keine Rückschlüsse in Bezug auf gestellte Diagnosen zu, inwieweit Psychotherapie via Internet oder Telefon von Klient_innen favorisiert wird. Dasselbe trifft auf das Alter der Klient_innen zu.

### Unbewusste Prozesse

Auch wenn es letzten Endes eine bewusste Entscheidung sein sollte, mediengestützte Psychotherapie anzubieten, erscheint es sinnvoll sich genau den unbewussten Motiven für eine Ablehnung oder Annahme mediengestützter Psychotherapie zu widmen. Dies betrifft sowohl Psychotherapeut_innen, als auch Klient_innen. So könnten z. B. die Bindungserfahrungen sowohl von Klient_innen, aber auch von Psychotherapeut_innen Einfluss auf die Beziehungsgestaltung und in weiterer Folge auf die Wahl der Methode und damit auch auf die Bereitschaft zum Einsatz von Medien haben.

Die Bindungsforschung bestätigt die Bedeutsamkeit früher Interaktionen für die Entwicklung von Gesundheit, wobei zwischen sicherem, unsicher-vermeidendem, unsicher-ambivalentem und desorganisiertem Bindungsstil unterschieden wird (Ainsworth et al. 1978). Svenja Taubner und Kolleg_innen präsentierten eine Studie zur Kompetenzentwicklung von angehenden Psychotherapeuten_innen, in der die aktuelle mentale Verarbeitung (aversiver) früher Kindheitserfahrungen zu Beginn der Ausbildung untersucht wurden (Taubner et al. 2015). Psychotherapeut_innen in Ausbildung scheinen hinsichtlich aversiver Kindheitserfahrungen ähnlich belastet wie die Normalbevölkerung. Der Anteil sicherer Bindung lag bei 78 % der Teilnehmer_innen, wovon 21 % als erworben-sicher eingestuft wurden. Ein kleiner Anteil präsentiert sich als unsicher-vermeidend, was damit erklärt werden könnte, dass diese Personengruppe weniger häufig den Beruf Psychotherapeut_in wählt (ebd.).

Die Bindungsfähigkeit ist ein spezifisches Vermögen des Menschen, mit Sozialpartnern in längerdauernde emotionale Wechselbeziehung einzutreten. Die vorliegende Studie zeigt, dass zumindest zwei Psychotherapeut_innen auch ohne dringliche Notwendigkeit ausschließlich die Anwendung von Psychotherapie mittels Medien aufrechterhalten wollten, ohne auf die Bedürfnisse ihrer Klient_innen einzugehen. Fünf Psychotherapeut_innen (nicht in die Studie einbezogen) beharrten auf dem üblichen face to face setting. Weitere Forschungsarbeit, inwieweit unterschiedliche Bindungsmuster für die Bevorzugung einer Methode vorliegen, wäre jedenfalls für weitere Diskussionen von Interesse.

### Psychotherapie in Ausnahmezeiten

Der Gedanke „Dreimal hinsehen, einmal handeln … Langsam und fehlerlos ist besser als schnell und zum letzten Mal“ (Nadolny 1987) könnte hilfreich bei der Suche nach der Antwort auf die Frage „Psychotherapie per Internet, qualitativ gleichwertig oder nicht?“ sein. Die Frage, ob Psychotherapie via Internet mit Psychotherapie ohne mediale Vermittlung dem Wesen nach gleichzuhalten ist, bleibt unbeantwortet und ist auch nicht Frage dieser Studie, weitere Forschung dazu ist jedoch notwendig.

Jedenfalls zu beachten sind auch weiterhin die Rahmenbedingungen des Psychotherapiegesetzes, wie die Ausübung nach bestem Wissen und Gewissen unter Beachtung der Entwicklung der Erkenntnisse der Wissenschaft und des Erwerbs spezifischer Kenntnisse und Erfahrungen (Bundesrecht 2021). Sich dabei nur auf die Kenntnisse des Mediengebrauchs zu fokussieren ist sicher eine Engführung.

Auch die Verpflichtung über die besonderen Rahmenbedingungen bei mediengestützter Psychotherapie wie z. B. Aufklärung, Datensicherheit, geschützter Raum und die Verpflichtung spezifischer Dokumentation wäre zu gewährleisten.

Auch wenn das Thema „Finanzielle Aspekte – Einsparung von Kosten bei Internetpsychotherapie“ von den interviewten Psychotherapeut_innen im Wesentlichen ausgespart wurde, ist hinsichtlich der Einhaltung der Werberichtlinie (BMSGPK 2010) Selbstbeschränkung notwendig. Werben doch bereits Pychotherapeut_innen mit dem Angebot „Therapie via Internet“ auf ihrer Homepage.

Anderseits bietet die Möglichkeit in bestimmten begründbaren, reflektierten Situationen Psychotherapie mittels Medien durchzuführen, eine Verbesserung der Versorgung psychisch Kranker.

## Individuell in die Zukunft

Psychotherapie ist immer ein individuelles Beziehungsgeschehen, in dem auch die Beziehungserfahrungen der Psychotherapeut_innen selbst mitspielen. Therapeutische Präsenz ist eine Vorbedingung für die stattfindenden (inneren) Prozesse. „Die Vorbedingung für das Erleben therapeutischer Präsenz bestehen darin, dass Therapeuten überhaupt die Intention zum Präsent-sein in der Therapie haben und dass sie Präsenz auch in ihrem Alltagsleben kennen und Erfahrungen damit haben.“ (Stumm und Keil 2014). Präsenz beinhaltet die Empfänglichkeit für alle Erfahrungen sinnlicher Art mit Klienten und Klientinnen in einem Resonanzraum (Finger-Ossinger 2018).

Inwieweit virtuelle Erfahrungen in der Gesellschaft insgesamt die Wahrnehmung und Erfahrung für das reale Präsent-Sein bereits verändert haben, kann nur vermutet werden. Virtuelle Welten haben einen maßgeblichen Einfluss darauf gewonnen, wie wir unsere tatsächlichen, alltäglichen Lebensräume gestalten und erleben. Mit Hilfe von VR-Simulatoren werden inzwischen nicht nur Fachärzte geschult, sondern auch psychische Beschwerden wie Phobien und PTSD behandelt (Michely 2017). (Anm.: Virtual Reality, virtuelle Realität oder abgekürzt VR bezeichnet die dreidimensionale und interaktive Wahrnehmung computergenerierter, künstlicher Umgebungen mit Hilfe von speziellen Datenbrillen und -handschuhen). Es wächst auch eine ganze Generation heran, die immer weniger zwischen den unterschiedlichen Welten unterscheidet (Weinhardt 2013). Umso mehr ist es verwunderlich – wie die Covid-19 Krise zeigt – dass es vor allem auch jungen Menschen nicht genügt, ausschließlich virtuell in Verbindung zu sein, wie deren Zusammenkünfte trotz Ausgangsbeschränkungen während der Covid19-Krise an diversen öffentlichen Orten zeigen.

Die Entwicklungstrias aus AdressatInnen und Nutzungsmustern, Institutionellen Bedingungen und Methoden ist als zusammengefügte Reflexionsfigur gedacht …Gerade Onlineberatung ist aber durch ihre noch junge Geschichte und der immanent eingebauten Verführung durch beeindruckende technologische Verbesserungen des Internet möglicherweise mehr als andere Hilfeformen Sozialer Arbeit gefährdet, in oberflächlichen Diskursen gefangen zu bleiben … Beständig neu entwickelten Kommunikationsdienste und Anwendungen gaukeln oft methodische Beorderungen vor, die nicht automatisch zu innovativen und verbesserten Verfahren führen, nur weil sie neu und schick sind. (ebd.).

Jedenfalls handelt es ich bei internetgestützten oder-basierten Verfahren nicht nur um eine Nutzung neuer Technologien, sondern es handelt sich um eigenständige Methoden (Gahleitner und Peschl 2016). Inwieweit diese Methoden aber jemals auch als „Psychotherapie“ bezeichnet werden dürfen, ist weiterhin ungeklärt.

Die in dieser Studie vorliegende unterschiedliche Annahme von mediengestützter Psychotherapie – die von den Krankenkassen für die Zeit der Pandemie bezahlt wird – durch Klient_innen (unabhängig vom Alter) kann ein Hinweis dafür sein, dass ein individuelles Herangehen immer im Fokus einer Psychotherapie stehen sollte und dass Psychotherapeut_innen angehalten sind, diese in Eigenverantwortung reflektiert und abseits von Eigeninteressen, zu gewährleisten. Die zu stellende und gleichzeitig nicht zu beantwortende Frage ist: „Was ist das *Optimum* für diesen konkreten Menschen“ und „ist das* Optimum *auch in einer Zeit nach Covid19 gewährleistet?“

Da sich die zugrundeliegenden Daten und die Auswertung vor allem auf die Zeit während der Pandemie beziehen, bleibt die Grundsatzfrage nach dem Wesen der Psychotherapie (Jaspers 1955; Gerhardinger 2020) und vor allem ob dieses Wesen mittels Einsatzes von Medien erhalten bleiben kann, unbeantwortet. Die Krankenkassen haben durch ihr pragmatisches Vorgehen gewährleistet, dass der Kontakt mit Patient_innen während der Pandemie honoriert aufrechterhalten werden konnte. Aufgrund geschaffener Tatsachen während eines Ausnahmezustandes erübrigt sich jedoch nicht weiterführende Forschung, ob internetgestützte Kontakte einer psychotherapeutischen Behandlung im herkömmlichen, direkt persönlichen Kontakt gleichzuhalten sind, und ob die gewährte Ausnahmesituation im Zeichen der Pandemie zum Regelfall werden soll.

Abschließend lässt sich festhalten, dass für den Einsatz von mediengestützter Psychotherapie, vorausgesetzt diese wird mit „persönlicher und unmittelbarer Berufsausübung“ (BMSGPK 2020) gleichzuhalten sein und vorausgesetzt eine *optimale *Gesundheitsversorgung soll gewährleistet werden, noch Forschungsarbeit zu leisten ist. Vor allem in den Bereichen: Wirksamkeit mediengestützter Psychotherapie (auch methodenspezifisch), Begriffsklärung und Aufklärung der Konsument_innen, Ausnahmen bei gewissen Diagnosen und im Blick auf die Person des/der Psychotherapeut_in als Wirkfaktor (Jaspers 1955) mit besonderer Beachtung der psychotherapeutischen Beziehung (Horvath et al. 2011) unter geänderten Bedingungen und Anforderungen.
